# Deep learning-based computed tomography image segmentation and volume measurement of intracerebral hemorrhage

**DOI:** 10.3389/fnins.2022.965680

**Published:** 2022-10-03

**Authors:** Qi Peng, Xingcai Chen, Chao Zhang, Wenyan Li, Jingjing Liu, Tingxin Shi, Yi Wu, Hua Feng, Yongjian Nian, Rong Hu

**Affiliations:** ^1^Department of Digital Medicine, School of Biomedical Engineering and Imaging Medicine, Army Medical University, Third Military Medical University, Chongqing, China; ^2^Department of Neurosurgery, First Affiliated Hospital,Southwest Hospital, Army Medical University, Third Military Medical University, Chongqing, China; ^3^School of Basic Medicine, Army Medical University, Third Military Medical University, Chongqing, China

**Keywords:** deep learning, intracerebral hemorrhage, computed tomography, segmentation, volume measurement

## Abstract

The study aims to enhance the accuracy and practicability of CT image segmentation and volume measurement of ICH by using deep learning technology. A dataset including the brain CT images and clinical data of 1,027 patients with spontaneous ICHs treated from January 2010 to December 2020 were retrospectively analyzed, and a deep segmentation network (AttFocusNet) integrating the focus structure and the attention gate (AG) mechanism is proposed to enable automatic, accurate CT image segmentation and volume measurement of ICHs. In internal validation set, experimental results showed that AttFocusNet achieved a Dice coefficient of 0.908, an intersection-over-union (IoU) of 0.874, a sensitivity of 0.913, a positive predictive value (PPV) of 0.957, and a 95% Hausdorff distance (HD95) (mm) of 5.960. The intraclass correlation coefficient (ICC) of the ICH volume measurement between AttFocusNet and the ground truth was 0.997. The average time of per case achieved by AttFocusNet, Coniglobus formula and manual segmentation is 5.6, 47.7, and 170.1 s. In the two external validation sets, AttFocusNet achieved a Dice coefficient of 0.889 and 0.911, respectively, an IoU of 0.800 and 0.836, respectively, a sensitivity of 0.817 and 0.849, respectively, a PPV of 0.976 and 0.981, respectively, and a HD95 of 5.331 and 4.220, respectively. The ICC of the ICH volume measurement between AttFocusNet and the ground truth were 0.939 and 0.956, respectively. The proposed segmentation network AttFocusNet significantly outperforms the Coniglobus formula in terms of ICH segmentation and volume measurement by acquiring measurement results closer to the true ICH volume and significantly reducing the clinical workload.

## Introduction

Intracerebral hemorrhage (ICH) is a hemorrhage caused by primary, non-traumatic vascular rupture in the brain parenchyma. In China, the incidence rate of ICH is approximately 69.6/100,000 people every year (Gbd 2016 Stroke Collaborators., 2019), and the mortality rate is 40% within 30 day. Only 12–39% of patients achieve long-term functional independence (Zhang et al., 2021), placing an enormous burden on society and families. For this reason, early and accurate judgment of the severity of an ICH is vital for guiding clinical treatment decisions and predicting the long-term outcomes of patients.

The larger the hematoma volume is, the worse the damage to brain tissue. In general, emergency intervention, including intubation, ventilation and neuromonitoring, should be applied for acute ICH (Sheng et al., 2022), and a surgical decision should be made if the hematoma volume exceeds 30 mL (Rodriguez-Luna et al., 2017). Therefore, accurate measurement of ICH volume is of vital significance for determining the severity of brain injuries. Computed tomography (CT), the preferred examination for the clinical diagnosis of ICHs, is convenient and fast and has a definite effect. Currently, the ICH volume is mostly measured *via* the “Coniglobus formula” in clinical practice (Kwak et al., 1983). The principle is to idealize the shape of the ICH as an ellipsoid and calculate its volume using the formula *V* = *A* × *B* × *C* × 1/2, where *V* is the ICH volume, *A* is the largest diameter of the lesion on the maximum ICH slice in the CT image, *B* is the largest width perpendicular to *A* in this slice, and *C* is the number of ICH slices × slice thickness. The “Coniglobus formula” is a simple and fast method for volume measurement and has acceptable accuracy for ellipsoid shaped ICHs. However, the non-ellipsoid shape of most ICHs in clinical practice and the limited experience of radiologists result in large errors in the measurement of ICH volume (Huttner et al., 2006; Freeman et al., 2008), thus producing a certain degree of uncertainty in clinical decisions. The quantitative CT method refers to the accurate slice-by-slice delineation of the hemorrhage site and calculation of the volume in a CT image and is regarded as the gold standard for non-invasive measurement of the ICH volume (Yan et al., 2013). However, this method is complex and time-consuming, making it difficult to apply in clinical practice.

With the flourishing development of artificial intelligence technology represented by deep learning (DL) in recent years, DL-based segmentation methods have been widely applied in the segmentation and measurement of brain tissues (Valliani et al., 2019). For ICHs, Cho et al. (2019) constructed a cascaded DL model for ICH detection and segmentation, with an accuracy of only 80.19% in ICH segmentation. Chilamkurthy et al. (2018) developed a DL model combined with natural language processing, trained it with over 300,000 brain CT images, and employed it for the identification of various subtypes of ICHs, skull fractures, and midline displacements (area under the receiving operator characteristic curve (AUC) >0.9). Ye et al. (2019) combined the convolutional neural network (CNN) VGG-16 and the bidirectional gate recurrent unit (Bi-GRU) to form an end-to-end deep neural network (DNN), which was then trained and tested using 2,836 brain CT images from three hospitals, with an AUC exceeding 0.8 in terms of the identification of different subtypes of ICHs. An end-to-end DNN model for ICH classification and segmentation was proposed by Kuo et al. (2019) that demonstrated a near-expert level in the detection of ICH subtypes. Arbabshirani et al. (2018) collected approximately 50,000 brain CT images to validate the effectiveness of a 3D CNN in ICH detection. Chang et al. (2020) established a CNN-based method for ICH segmentation. Zhao et al. (2021) applied the nnU-Net framework to the segmentation and volume calculation of ICH and peripheral oedema. Xu et al. (2021) evaluated Dense U-Net framework for the segmentation and quantification of ICH, EDH (extradural hemorrhage) and SDH (subdural hemorrhage). Rava et al. (2021) evaluated the Canon automatic stroke detection system and the automatic ICH segmentation tool in Vitrea and investigated the performance of the system in ICH detection and the effect of ICH volume on the detection performance of the system. Compared with the Coniglobus formula, deep learning technology demonstrates a higher accuracy in measuring the intracerebral hematoma volume (Lai et al., 2020; Jia et al., 2021). However, most studies of ICH segmentation have focused on the application of existing deep networks, lack of improvement on deep network to further improve performance.

In this study, a large-scale dataset of ICH CT images was established, and a DLN (AttFocusNet) for accurate segmentation of CT images of ICH was constructed and compared with other typical DLNs in terms of segmentation performance. Experimental results in both internal and external validation sets showed that AttFocusNet outperformed other networks in terms of segmentation, exhibited much higher accuracy than the Coniglobus formula in terms of ICH volume measurement, and significantly reduced the workload of ICH volume measurement in clinical practice, thus offering strong support for the clinical diagnosis of ICH.

## Materials and methods

### Clinical data

The data used in this study were head CT images of 1,027 ICH patients within 24 h of admission to first affiliated hospital of Army Medical University (Southwest Hospital), with a CT slice thickness of 4.0 mm. All CT scans were randomly assigned to the training dataset, validation dataset and testing dataset at a ratio of 7:1:2 during the experiment. Table 1 shows the detailed statistical information of each dataset. Age and hematoma volume are reported as the mean ± standard deviation (SD), and the Glasgow coma scale (GCS) score and ICH score are reported as the median (minimum, maximum). In addition, ICHs were observed in the basal ganglia for approximately 76% of the cases and found in locations such as lobes for the rest of the case. If an ICH was found in multiple locations at the same time, it was classified according to the first ICH location in the pathology report.

**TABLE 1:** Description of the data features of the training set, validation set, and testing set.

Item	Training set	Validation set	Testing set	*P*-value
Number of cases (*n*)	718	102	207	
Age (Mean ± SD)	57.19 ± 12.67	55.56 ± 12.95	55.93 ± 13.33	0.211
GCS [Median (Min, Max)]	13 (3, 15)	13 (3, 15)	13 (5, 15)	0.896
ICH [Median (Min, Max)]	1 (0, 4)	1 (0, 4)	1 (0, 4)	0.664
Hemorrhage volume (Mean ± SD)	32.65 ± 22.97	33.10 ± 25.90	33.83 ± 20.19	0.323
Intraventricular extension of intracerebral hemorrhage (*n*)	277	40	78	
**Hemorrhage location**				
Basal ganglia	551	77	154	
Lobe	143	20	46	
Brain stem	5	0	1	
Cerebellum	3	0	1	
Ventricle	16	5	5	

GCS, Glasgow coma scale; ICH, intracerebral hemorrhage; SD, standard deviation.

The resolution of all CT images was resampled to a 512 × 512 matrix, and then the scan slices of chest and abdomen were removed. As a result, 40 slices of CT images of each patient were preserved. The CT values of ICHs were mainly concentrated in the range of [−60, 140]. To ensure the segmentation effect, before the CT images were sent to the DLN, they were first windowed according to the above CT value range and then normalized. This data collection was reviewed and approved by the Ethics Committee of First Affiliated Hospital of Army Medical University (Southwest Hospital) (No. KY2021185).

### Development of the deep learning model for intracerebral hemorrhage segmentation

**FIGURE 1:**
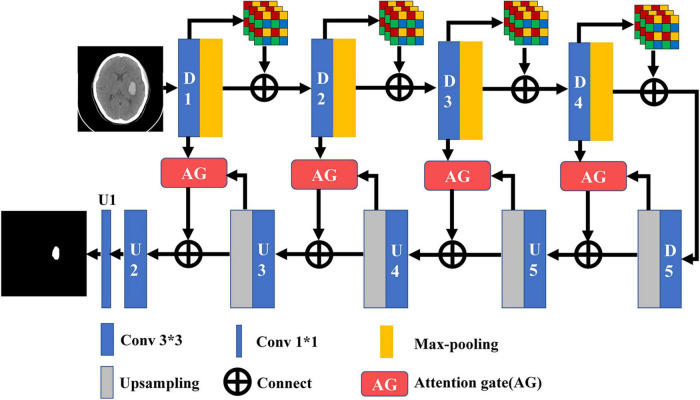
The structure diagram of AttFocusNet based on focus and the AG (D* and U* represent the convolutions in the encoding process and decoding process, respectively).

The attention gate (AG) is an attention mechanism proposed by Oktay et al. (2018) for three-dimensional (3D) segmentation of pancreatic CT images. In the model learning stage, the AG is able to suppress the parts irrelevant to the task features and focus on learning the features related to the task. In this study, the two-dimensional (2D) implementation of the AG is carried out and then combined with U-Net to obtain a 2D segmentation network, AttUNet. On this basis, a 2D segmentation network, AttFocusNet, based on AttUNet and the focus structure is proposed, which can be seen in Figure 1. The CT images of ICHs are input slice by slice, and the image features are extracted using an encoder with a focus structure, which is combined with AG in the encoder stage, to finally obtain the accurate segmentation of ICHs.

**FIGURE 2:**
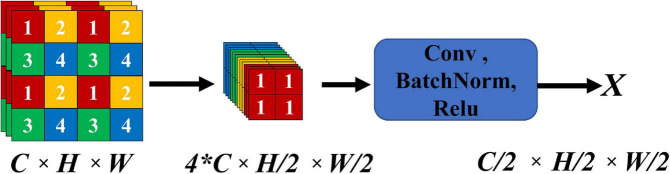
The scheme of the focus structure.

In DL-based image segmentation methods, feature dimensionality reduction is performed by encoders mostly through convolutional pooling. The signals at some small ICH points are hypointense on CT images, so they may be deemed to be redundant information and removed during feature extraction. The focus structure in YOLO-V5 is introduced to integrate the image information into the channel space for convolution, and the complete image features without pooling are fused with the features after convolutional pooling to effectively preserve the integrity of the overall features of the ICH during encoding. Figure 2 shows the focus structure, where *C*, *H*, and *W* represent the number of channels, height and width, respectively. In the focus structure, a feature map with *C* channels was sampled with a step size of 2 to evenly distribute the features of the feature map into 4*C* channels, and the feature map with *C*/2 channels was then output through convolution.

**FIGURE 3:**
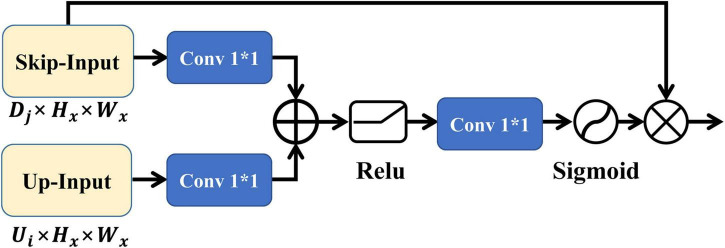
The structure diagram of 2D AG.

The output of the focus structure contains redundant low-level features that can be effectively suppressed by the AG, thus capturing more important features relevant to the task. The 2D implementation of the AG is shown in Figure 3, where the inputs skip-input and up-input have the same feature size, where up-input is the feature input after sampling on the *U*_*i*_ slice and skip-input is the feature input of the *D*_*j*_ slice of the encoder corresponding to the *U*_*i*_ slice. After convolution, up-input and skip-input are subjected to feature splicing, pass through the rectified linear unit (ReLU) activation function, and are subjected to the next convolution. Finally, the features of the sigmoid activation function are integrated with those of skip-input to obtain the output of the 2D AG.

The proposed AttFocusNet was implemented based on the PyTorch DL framework, with a workstation equipped with NVIDIA Quadro RTX5000 video memory (16 G) for model training and the RMS prop optimization function as the optimizer. The initial learning rate, epoch and batch size were set to 0.00001, 40 and 2, respectively. The learning rate was adjusted by a conditional trigger strategy, which was triggered when the model failed to converge for two consecutive epochs.

### Volume measurement

The manual segmentation results of CT slices obtained by neurosurgeons were considered as the ground truth (GT), which could be used to evaluate the segmentation performance of deep models. The segmentation of each CT slice in this study was performed by a junior neurosurgeon with 7 years of working experience using the Mimics software, and the segmentation result was further corrected by a senior neurosurgeon with 20 years of working experience. From the Digital Imaging and Communications (DICOM) file header of CT images of ICHs, the voxel spacing *X* and *Y* in the horizontal and vertical directions, respectively, as well as the slice thickness *T* of CT scan can be obtained. Once the ground truth of all slices was obtained, the intracerebral hemorrhage volume of each patient could be calculated as follows


(1) $$V=\sum_{i=1}^{L}X\,Y\,T\,W_{i}$$

where *W*_*i*_ is the total number of voxels in the *i*(1 ≤ *i* ≤ *L*)-th slice of the ICH area and *L* is the total number of slices of the CT image. Note that the intracerebral hemorrhage volume that calculated using GT of each slice could be considered as the ground truth of intracerebral hemorrhage volume (V-GT).

### Evaluation of performance

The ICH segmentation performance was evaluated using the Dice, intersection-over-union (IoU), sensitivity, positive predictive value (PPV), and 95% Hausdorff distance (HD95) metrics. Let true positive (TP) represent the number of true positive samples predicted to be positive samples, false positive (FP) represent the number of true negative samples predicted to be positive samples, and false negative (FN) represent the number of true positive samples predicted to be negative samples. Then, the above indicators are calculated as follows.


(2) $$D\,i\,c\,e=\frac{2\,T\,P}{F\,P+2\,T\,P+F\,N}$$

(3) $$I\,o\,U=\frac{T\,P}{F\,P+T\,P+F\,N}$$

(4) $$S\,e\,n\,s\,i\,t\,i\,v\,i\,t\,y=\frac{T\,P}{T\,P+F\,N}$$

(5) $$P\,P\,V=\frac{T\,P}{T\,P+F\,P}$$

Note that HD represents the distance between the surface point sets of the calculated true sample and the predicted sample. Let *G* denote the ground truth and *P* denote the predicted value set of ICH. HD can be calculated as follows:


(6) H⁢D⁢(G,P)=max⁡(h⁢(G,P),h⁢(P,G))

where $h\,\left(G,P\right)=\max_{g\in G}\left\{\min_{p\in P}||g-p||\right\}$ and $h\,\left(P,G\right)=\max_{p\in P}\left\{\min_{g\in G}||p-g||\right\}$

Note that the HD value is usually multiplied by 95% (HD95) for practical application to eliminate the influence of a very small subset of outliers.

## Results

### Metrics evaluation

AttFocusNet was compared with UNet++ (Zhou et al., 2020), AttUnet, PraNet (Fan et al., 2020), 3DUNet (Çiçek et al., 2016), and UNETR (Hatamizadeh et al., 2022) in terms of segmentation performance (Table 2). AttFocusNet exhibited the best comprehensive performance, outperforming other networks in terms of Dice, IoU, sensitivity, PPV, and, more prominently, HD95, fully demonstrating the effectiveness of AttFocusNet.

**TABLE 2:** Comparison of the segmentation performance of different methods.

Algorithm	Dice	IoU	Sensitivity	PPV	HD95 (mm)
UNet ++	0.900	0.865	0.911	0.950	6.829
AttUNet	0.893	0.859	0.899	0.956	7.253
PraNet	0.788	0.716	0.795	0.905	16.01
3DUNet	0.857	0.806	0.866	0.930	6.873
UNETR	0.700	0.638	0.825	0.799	13.668
AttFocusNet	0.908	0.874	0.913	0.957	5.960

**FIGURE 4:**
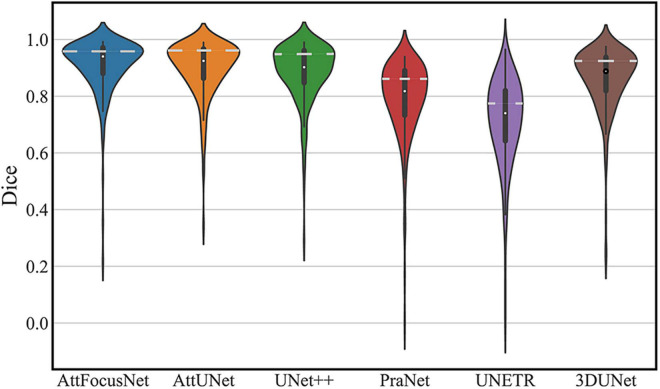
Distribution of the segmentation performance of different deep models (White dots, squares, vertical lines, and peaks represent the median, interquartile range, 95% confidence interval, and data density distribution, respectively).

The segmentation performance of each network on the testing dataset is shown in a violin plot (Figure 4), where the width between the left and right peaks (as shown by the white dashed lines) indicates the density of the data distribution.

### Segmentation effect

**FIGURE 5:**
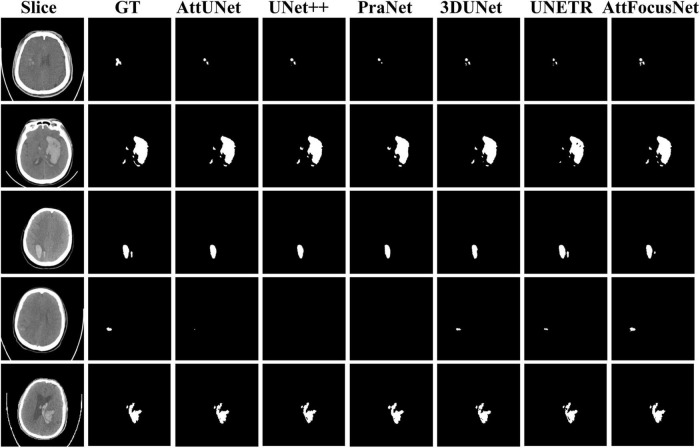
Comparison of the segmentation results of different methods.

Figure 5 exhibits segmentation results from the different methods. The first row shows the original CT image of different slices, the second row shows the corresponding GT with respect to each slice in the first row, and rows 3–8 show the segmentation results by different methods.

**FIGURE 6:**
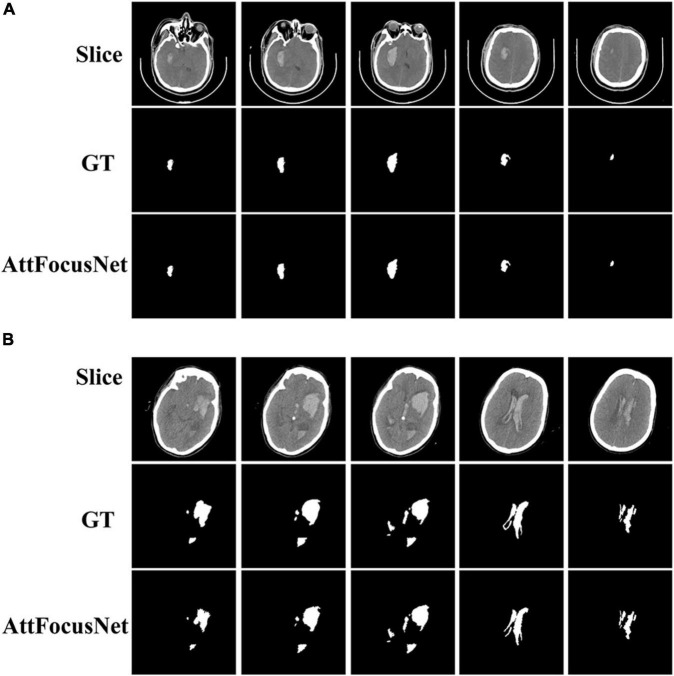
Computed tomography (CT) images and segmentation results corresponding to the difference in volume measurements between AttFocusNet and the Coniglobus formula. **(A)** A difference of 1.86 mL between volume measurements. **(B)** A difference of 74.05 mL between volume measurements.

Figure 6 presents the CT images and segmentation results of the patients for whom the results of the two methods show differences of 1.86 and 74.05 mL, respectively.

**FIGURE 7:**
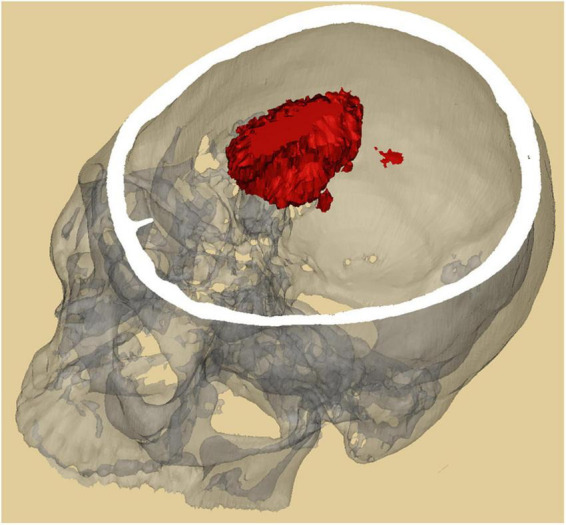
Three-dimensional (3D) visualization of an ICH by mimics.

The segmentation results obtained by AttFocusNet were used to generate the 3D visualization of the ICH *via* Mimics software, as shown in Figure 7.

### Consistency evaluation

**FIGURE 8:**
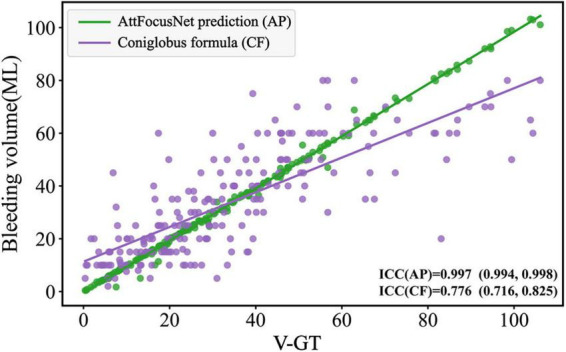
Comparison of the consistency.

The consistency of different methods was evaluated by linear regression as shown in Figure 8, where the abscissa scale is the V-GT and the ordinate represents the ICH volume measured by the different methods.

### External validation

In order to further evaluate the effectiveness of AttFocusNet, we, respectively, selected CT images of 50 patients from CQ500 dataset (Chilamkurthy et al., 2018) and RSNA2019 dataset (Flanders et al., 2020) for external validation, which was shown in Tables 3, 4. The results showed that AttFocusNet outperformed the other deep models on most metrics, fully demonstrating the effectiveness of AttFocusNet.

**TABLE 3:** Comparison of the segmentation performance of different methods on CQ500 dataset.

Algorithm	Dice	IoU	Sensitivity	PPV	HD95 (mm)
UNet ++	0.883	0.790	0.826	0.947	13.584
AttUNet	0.879	0.783	0.812	0.957	11.312
PraNet	0.809	0.680	0.747	0.882	24.161
3DUNet	0.842	0.727	0.782	0.913	37.052
UNETR	0.714	0.555	0.604	0.873	69.893
AttFocusNet	0.889	0.800	0.817	0.976	5.331

**TABLE 4:** Comparison of the segmentation performance of different methods on RSNA2019 dataset.

Algorithm	Dice	IoU	Sensitivity	PPV	HD95 (mm)
UNet ++	0.895	0.810	0.834	0.965	3.735
AttUNet	0.896	0.811	0.829	0.974	4.733
PraNet	0.775	0.633	0.647	0.966	6.842
3DUNet	0.820	0.694	0.707	0.975	10.838
UNETR	0.361	0.220	0.572	0.264	133.924
AttFocusNet	0.911	0.836	0.849	0.981	4.220

**FIGURE 9:**
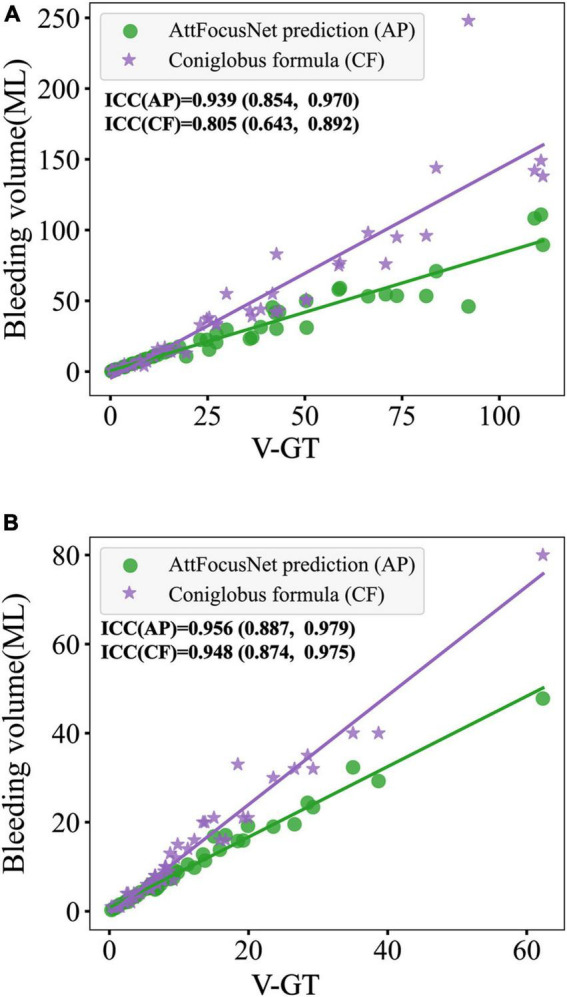
Comparison of the consistency for external validation. **(A)** CQ500. **(B)** RSNA2019.

Similar with Figure 8, the consistency of different methods in external validation set was evaluated by linear regression as shown in Figure 9, where the ICC of the ICH volume measurement on CQ500 and RSNA2019 between AttFocusNet and the ground truth were, respectively, 0.939 and 0.956, while the ICC of the ICH volume measurement between Coniglobus formula and the ground truth were, respectively, 0.805 and 0.948.

## Discussion

A major objective of our study was to accurately segment the ICH area from CT scans by DL as the premise of accurate volume calculation. We constructed large-scale CT scans of ICH, which is very important to ensure the segmentation performance of the DL model. The proposed AttFocusNet combines the focus structure with AttUNet. AttFocusNet outperformed the other five DL models in five indicators, especially HD95, clearly illustrating its effectiveness for ICH segmentation. AttFocusNet segments the ICH area from CT scans slice by slice and is significantly superior to 3D DL models in the overall segmentation of ICH from CT scans. The difficulty of training a 3D segmentation model is significantly higher than that of a 2D segmentation model, while the segmentation efficiency of a 3D DL model is usually lower than that of a 2D DL model. Therefore, the use of slice-by-slice mode in AttFocusNet can be considered a good choice for the volume measurement of clinical ICH. As shown in Figure 4, the Dice coefficient distributions of AttUNet and UNet + were between 0.8 and 1.0; however, the significantly broader width indicated that AttUNet achieved better comprehensive performance on the testing dataset. The Dice coefficient distributions of PraNet, 3D U-Net and UNETR were mainly between 0.7 and 0.9 with a narrow width, indicating poor segmentation performance. AttFocusNet achieved the best performance with a Dice coefficient close to 1.0 and the broadest width, indicating excellent segmentation performance.

As shown in Figure 5, regardless of whether the bleeding area was large or small, or the bleeding shape was ellipsoid or non-ellipsoid, AttFocusNet always achieved the best segmentation performance among the evaluated DL models, thus providing a good foundation for the calculation of ICH volume. As shown in Figure 6A, when the ICH had a relatively ellipsoid shape, the volume measured by the Coniglobus formula was similar to that measured by AttFocusNet. When the ICH had a highly non-ellipsoid shape, as shown in Figure 6B, the volume measurements of the Coniglobus formula and AttFocusNet differed considerably, and the volume measured by the Coniglobus formula was less accurate. The above results are completely consistent with our understanding of the Coniglobus formula. In fact, if the shape of ICH is non-ellipsoidal, compared with the Coniglobus formula, AttFocusNet was essentially validated against a poor method. Note that most ICHs are non-ellipsoid in shape, which means that our method has better application prospects in clinical practice. The ICH score (Hemphill et al., 2001) is a strong predictor of 30-day mortality and includes five independent indicators: GCS score, age, ICH volume, IVH and ICH origin. This information can be obtained from CT scans with the exception of the GCS score, which must be evaluated by clinicians. The proposed AttFocusNet is helpful for the volume measurement of ICH regardless of hematoma shape, which could be helpful to accurately determine the ICH score. Based on the segmentation results, we can easily establish a 3D model for the visualization of ICH by using Mimics software. 3D visualization can help clinicians conduct comprehensive and accurate observations and analyses of ICH from a three-dimensional perspective to design accurate treatment schemes and improve the level of medical diagnosis and treatment and the utilization value of medical imaging.

From the perspective of the fitting of the linear regression, the volumes measured by the Coniglobus formula were highly scattered, with a number of outliers, poor consistency with the V-GT, and an intraclass correlation coefficient (ICC) of 0.776, indicating weak consistency between the Coniglobus formula and the V-GT. In contrast, the volumes measured by AttFocusNet showed an ideal fit, with few outliers, high consistency with the V-GT, and an ICC of 0.997, indicating strong consistency between AttFocusNet and the V-GT.

In terms of efficiency, AttFocusNet took approximately 5.6 s to provide automatic segmentation and volume analysis for each patient, while the Coniglobus formula and manual segmentation by the neurosurgeon (based on the Mimics software) required 47.7 and 170.1 s for each patient on average. Therefore, AttFocusNet can reduce the clinical workload significantly while ensuring high measurement accuracy of ICH volume. Once the ICH segmentation results are obtained, we can accurately calculate the ICH volume. In terms of consistency evaluation, compared with the Coniglobus formula, the volume calculated by AttFocusNet was closer to the V-GT, which further shows the effectiveness of the segmentation algorithm.

Similar with internal validation, AttFocusNet outperformed the other deep models for the external validation, which showed that AttFocusNet had better generalization performance. Moreover, according to the ICC values, compared with the Coniglobus formula, the ICH volume obtained by AttFocusNet had better consistency with V-GT, which fully showed the effectiveness of the proposed deep network.

There were several limitations to our work. First, the hematoma subtypes of the patients were either ICH or IVH, while other subtypes, including extradural hemorrhage (EDH), subdural hemorrhage (SDH), and subarachnoid hemorrhage (SAH), were excluded. In addition, patients under 18 years of age were excluded, which could limit the generalizability of our model. Second, the ground truth masks required consensus from more than one human expert, which would reduce errors caused by fatigue, technical overload, lack of concentration and other factors.

In conclusion, AttFocusNet provided automatic segmentation and volume measurement of ICH. AttFocusNet achieved high accuracy in ICH volume measurement regardless of ICH shape and significantly reduced the clinical workload, indicating that AttFocusNet is a promising approach for clinical practice. At present, Transformer has been gradually applied to the feature extraction of 3D data in the construction of DLNs, and the combination of AttFocusNet and Transformer is expected to further improve the segmentation effect in the future.

## Data availability statement

The original contributions presented in this study are included in the article/supplementary material, further inquiries can be directed to the corresponding authors.

## Ethics statement

This study was approved by the Ethics Committee of First Affiliated Hospital of Army Medical University (Southwest Hospital) (No. KY2021185). Written informed consent was not required for the study on human participants in accordance with the local legislation and institutional requirements.

## Author contributions

YN, RH, and HF are responsible for the design of the study and review of the manuscript. QP and CZ contributed to data collection. XC and JL contributed to data analysis. QP, XC, and YN contributed to manuscript preparation. YW, WL, and TS contributed to manuscript revision. All authors have read and approved the final manuscript.
